# 511. Treatment with Molnupiravir in the MOVe-In and MOVe-Out Clinical Trials Results in an Increase in Transition Mutations Across the SARS-CoV-2 Genome

**DOI:** 10.1093/ofid/ofab466.710

**Published:** 2021-12-04

**Authors:** Julie Strizki, Jay Grobler, Ying Zhang, Jiejun Du, Shunbing Zhao, Diane Levitan, Alex Therien, Joan R Butterton, Nicholas Murgolo

**Affiliations:** Merck & Co., Inc., Kenilworth, New Jersey

## Abstract

**Background:**

Molnupiravir (MOV), (MK-4482, EIDD-2801) is being clinically developed for the treatment of COVID-19 disease caused by SARS-CoV-2. MOV is the orally administered 5′-isobutyrate prodrug of the active, antiviral ribonucleoside analogue, N-hydroxycytidine (NHC, EIDD-1931) which inhibits viral replication by induction of mutations in the viral genome, leading to viral error catastrophe. In 2 clinical studies, hospitalized (MOVe-In) and non-hospitalized (MOVe-Out) participants were treated for 5 days with MOV and followed up to Day 29. Viral RNA isolated from nasal swab samples were sequenced to determine the rate, distribution and type of viral mutations observed after MOV treatment.

**Methods:**

RNA isolated from nasopharangeal swab samples collected during study conduct was quantified by RT-PCR. Samples containing >22,000 copies/mL of RNA underwent complete genome NGS using the Ion AmpliSeq SARS-CoV-2 research panel and Ion Torrent sequencing. Mutation rates were calculated by determining the number of nucleotide changes observed across the entire genome at Day 3 and/or Day 5 compared to baseline.

**Results:**

Combined data from both studies showed an increase of ~2-4 fold in the viral mutation rate post-baseline in MOV treated compared with placebo. Mutations were distributed across the entire genome with only a minority being observed in more than one sample. The most frequent mutations were transitions of C to U observed in the highest MOV dose group (800 mg/BID).

**Conclusion:**

Consistent with the proposed mechanism of action of MOV, an increase in the rate of transition mutations in the virus was observed in post-baseline nasal swab samples from participants treated with MOV compared with placebo.

**Disclosures:**

**Julie Strizki, PhD**, **Merck & Co., Inc.** (Employee, Shareholder) **Jay Grobler, PhD**, **Merck & Co., Inc.** (Employee, Shareholder) **Ying Zhang, PhD**, **Merck & Co., Inc.** (Employee, Shareholder) **Jiejun Du, PhD**, **Merck & Co., Inc.** (Employee, Shareholder) **Shunbing Zhao, PhD**, **Merck & Co., Inc.** (Employee, Shareholder) **Diane Levitan, PhD**, **Merck & Co., Inc.** (Employee, Shareholder) **Alex Therien, PhD**, **Merck & Co., Inc.** (Employee, Shareholder) **Joan R. Butterton, MD**, **Merck Sharp & Dohme Corp.** (Employee, Shareholder) **Nicholas Murgolo, PhD**, **Merck & Co., Inc.** (Employee, Shareholder)

